# Local-Scale DNA Barcoding of Afrotropical Hoverflies (Diptera: Syrphidae): A Case Study of the Eastern Free State of South Africa

**DOI:** 10.3390/insects14080692

**Published:** 2023-08-04

**Authors:** Michel Mathurin Kamdem, Mpho Ramoejane, Patricks Voua Otomo

**Affiliations:** Department of Zoology and Entomology, University of the Free State, Private Bag x13, Phuthaditjhaba 9866, South Africa; ramoejanem1@ufs.ac.za (M.R.); otomopv@ufs.ac.za (P.V.O.)

**Keywords:** Afrotropical hoverflies, COI gene, species delimitation, genetic richness, local-scale studies

## Abstract

**Simple Summary:**

Hoverflies are regarded as the second most important pollinators after bees. They also provide important environmental services including the biodegradation of organic wastes, as well as the predation of pests. Hoverflies are usually divided into several groups or regions including the Holarctic, the Oriental, the Australasian and the Afrotropical. The latter is considered one of the most diverse groups but is still poorly studied due to the unavailability of complete and detailed identification keys for numerous genera and/or species. Published taxonomy studies on hoverflies in South Africa were published in the 1980s. This study aimed to investigate the barcoding of hoverfly species found in the Free State province of South Africa in order to ascertain their taxonomy and establish their genetic richness and differentiation. From 78 specimens of hoverflies sampled in the eastern Free State of South Africa, DNA barcodes helped to confirm the taxonomy of 15 hoverfly species from nine genera. With the barcodes generated in this study, the identification of Afrotropical species can be improved, but about 40% of the known species cannot be identified using the available identification keys.

**Abstract:**

The Afrotropical hoverflies remain an understudied group of hoverflies. One of the reasons for the lack of studies on this group resides in the difficulties to delimit the species using the available identification keys. DNA barcoding has been found useful in such cases of taxonomical uncertainty. Here, we present a molecular study of hoverfly species from the eastern Free State of South Africa using the mitochondrial cytochrome-c oxidase subunit I gene (COI). The identification of 78 specimens was achieved through three analytical approaches: genetic distances analysis, species delimitation models and phylogenetic reconstructions. In this study, 15 nominal species from nine genera were recorded. Of these species, five had not been previously reported to occur in South Africa, namely, *Betasyrphus inflaticornis* Bezzi, 1915, *Mesembrius strigilatus* Bezzi, 1912, *Eristalinus tabanoides* Jaennicke, 1876, *Eristalinus vicarians* Bezzi, 1915 and *Eristalinus fuscicornis* Karsch, 1887. Intra- and interspecific variations were found and were congruent between neighbour-joining and maximum likelihood analyses, except for the genus *Allograpta* Osten Sacken, 1875, where identification seemed problematic, with a relatively high (1.56%) intraspecific LogDet distance observed in *Allograpta nasuta* Macquart, 1842. Within the 78 specimens analysed, the assembled species by automatic partitioning (ASAP) estimated the presence of 14–17 species, while the Poisson tree processes based on the MPTP and SPTP models estimated 15 and 16 species. The three models showed similar results (10 species) for the Eristalinae subfamily, while for the Syrphinae subfamily, 5 and 6 species were suggested through MPTP and SPTP, respectively. Our results highlight the necessity of using different species delimitation models in DNA barcoding for species diagnoses.

## 1. Introduction

More than 6000 species of hoverflies have been recorded worldwide, with approximately 600 species from the Afrotropical region distributed into three subfamilies, namely, Eristalinae, Microdontinae and Syrphinae [1,2,3]. Adult hoverflies (Syrphidae family) are often regarded as one of the most important pollinator groups of the Diptera order after bees [4,5,6]. This important ecosystem service that they provide in both natural and agricultural land uses makes hoverflies a key model group for impact surveys of agricultural and horticultural processes as well as pest management [5,7,8,9].

One critical issue for these applications is that the understanding of the response of hoverflies to landscape management in relation to their diversity can be challenged by the limitation of the usable identification keys for Afrotropical species [2]. This has been particularly highlighted in the genus *Eumerus* Meigen, 1822, which has been shown to be too vague for species identification through the routine use of morphological taxonomy tools [10]. Most of these Afrotropical species were described centuries ago, and their morphological diagnoses have been used in various studies without thorough revisions to encompass as much species diversity as found within the region [11,12]. Although morphological diagnoses have their limitations, the method is very useful when used with molecular techniques [13]. This indicates the need to use an informative and effective molecular technique to assist in morphological identification as a prerequisite for efficient conservation [13].

DNA barcoding [14] is currently the most used molecular taxonomy tool that provides the possibility of distinguishing animal species by sequencing a fragment of the 5′ end of the COI (cytochrome c oxidase subunit 1) mitochondrial gene. In addition to the detection of sequence variation within and between taxa, the use of this approach holds many other advantages in the study of Afrotropical hoverflies. It is recommended for those taxa that deserve further taxonomic investigations due to ambiguous morphology [15]. It links the unknown sex to species for which only one of the sexes is known, thus reducing bias in species-level data [15]. Moreover, DNA barcoding can be used to relate the developmental stages of species for which the reproductive ecology is poorly understood [15]. Afrotropical hoverflies constitute a large and diverse group [6,16], but few studies have focused on the genetic diversity of this group [11,17,18]. It is estimated that a major part of the existing species of Afrotropical hoverflies has not been described to date [2,16,17,18,19,20]. The occurrence of cryptic species may also complicate species identification. In their study, Jordaens et al. [17] reported the possibility of the occurrence of cryptic species in Afrotropical hoverflies and observed that differentiating intra- from interspecific divergence among subfamilies using distance analyses was better defined at the genus level. That study remains the most important contribution to the study of Afrotropical hoverflies both from a morphological and a DNA barcoding perspective. More recently, many researchers have made substantial contributions to species delimitations and phylogenetic inferences in the *Merodon* Meigen, 1803 [21,22], *Sphaerophoria* Le Peletier & Serville, 1828 [20] and *Eumerus* [18,19] genera of Afrotropical hoverflies.

In South Africa, hoverfly species have been studied based on their external morphology, and the country harbors species from three out of the four hoverfly subfamilies, namely, Syrphinae, Microdontinae and Eristalinae [23]. The need for molecular studies on these beneficial flies is critical to conclusively establish species diversity and genetic differentiation between subfamilies. This study aimed to conduct a DNA barcoding investigation of hoverfly species from selected areas of the eastern Free State of South Africa in order to ascertain their taxonomy and establish their genetic richness and differentiation.

## 2. Materials and Methods

### 2.1. Field Sampling, DNA Extraction and Sequencing

**Figure 1:**
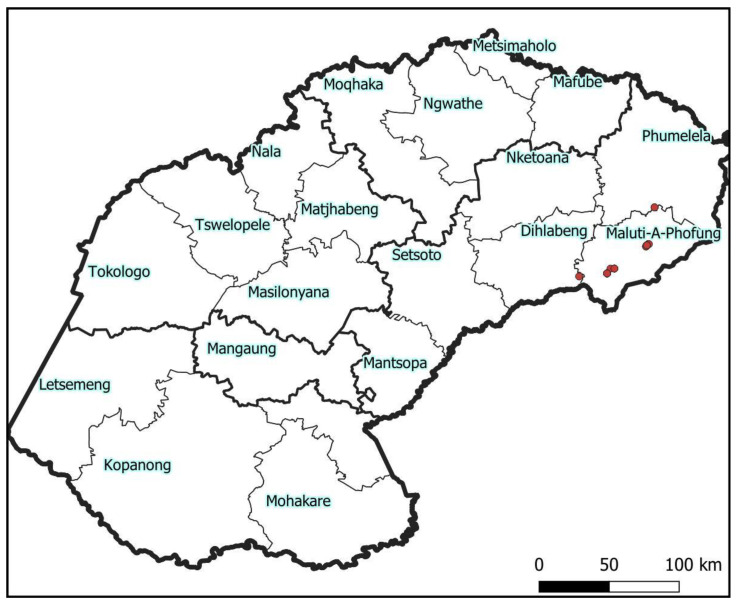
Study area in the Maluti-A-Phufong and Phumelela municipalities (Free State province of South Africa). Red circles represent sampling locations.

Specimens of hoverflies were collected using entomological nets. The sampling ensured the inclusion of as much species diversity as possible from the study area, which encompassed the towns of Harrismith (28°17′0″ S 29°08′0″ E) and Phuthaditjhaba (28°32′00″ S 28°49′00″ E), the Golden Gate Highlands National Park (28°30′22″ S 28°37′0″ E) and the farming lands near the village of Verkykerskop (27.918° S 29.281° E) in the Maluti-A-Phufong and Phumelela municipalities (Figure 1). Collected specimens were sorted into morphotypes using the descriptions and illustrations in Whittington [2], Jordaens et al. [17] and Scholtz and Holm [23], preserved in 99% ethanol and deposited in the museum of the Department of Zoology and Entomology of the University of the Free State-QwaQwa Campus.

Total genomic DNA was extracted from two legs of each individual following the method described by Mengual et al. [24]. This was done following the instructions of the manufacturer of the NucleoSpin^®^ Tissue DNA Extraction kit (Macherey-Nagel, Düren, Germany). Tissue samples were pre-lysed in the presence of Proteinase K at 37 °C for 10 h with vortexing every 1 h. Thereafter, buffer B3 from the NucleoSpin ^®^ Tissue DNA Extraction kit was added to the lysed samples and incubated at 70 °C for 10 min. DNA was eluted using 100 µL buffer BE at room temperature. The eluted DNA was stored at −35 °C until PCR could be performed. A negative control (blank) without tissue was included in each set of DNA extractions. A total of 108 specimens were processed for DNA extraction.

Each reaction tube included the following components: 1–3 µL DNA extract, 1 µL of each primer (with an initial concentration of 10 μM), 9.5 μL nuclease-free water and 12.5 μL OneTaq^®^ 2X Master Mix (Inqaba Biotechnical Industries, Pretoria, Gauteng, South Africa) with Standard Buffer. OneTaq^®^ 2X Master Mix is a ready-to-use PCR master mix that also contains agarose gel loading buffer and tracking dyes. The universal primers were used to amplify 574 bp of the COI gene (Table 1). Amplifications were performed in 0.2 mL thin-walled PCR tubes using the Multigene Optimax thermocycler (Labnet International, Inc, New York, NY, USA). PCR conditions included an initial denaturation at 95 °C for 2 min, followed by 29 cycles for 30 s, denaturation at 94 °C for 30 s, annealing at 49 °C for 2 min and extension at 72 °C, followed by a final extension for 8 min at 72 °C [24]. Negative controls consisting of the PCR reaction mix excluding the DNA extract were also used to control for DNA contamination. Successful amplification was checked based on size and products (bands) by running the PCR product (2 µL) on an agarose gel (0.5 g SeaKem^®^ LE Agarose (Lonza Rockland, Inc., Rockland, ME, USA), Lonza in 50 mL TAE buffer, 1.5% *w*/*v*) stained with 2 μL ethidium bromide. Specifically, 2 μL of the PCR product was loaded and run for 30 min at 100 V and 350 Ma. The gel was visualized using the Gene genius Bioimaging System and SynGene software (Gensnap v6.00.22. 1., Synoptics, Ltd., (Synoptics, Ltd., Cambridge, UK). The samples were then shipped to Inqaba biotec^TM^ (Hatfield, Pretoria, South Africa) for one-directional DNA sequencing with the LCO1490 primer. Sequencing reactions were performed using the ABI v3.1 BigDye^®^ kit (Applied Biosystems, Waltham, MA, USA). Purified sequences were run on an ABI 3500 XL Genetic analyzer, POP7TM (ThermoScientific, Waltham, MA, USA).

### 2.2. Sequencing Analysis

The sequences (574 bp) were screened, cleaned and edited using SeqMan Ultra (DNASTAR Inc., Madison, WI, USA). After low-quality sequences were removed, 78 were retained out of the 108 sequences generated. These sequences were deposited in Genbank (see the Table A1 in Appendix A for the accession numbers). They were tentatively identified using the BOLD (Barcode of Life Data Systems) identification system and compared to published COI sequences of Afrotropical hoverflies deposited in Genbank by Jordaens et al. [17] and other sources. All sequences were queried through Blast search (BLAST v.2.9) using blastn in the nr/nt database in NCBI’s Genbank. Sequences were translated to protein using the invertebrate mitochondrial code in MEGA X [26]. The sequences were aligned in MUSCLE and trimmed in length in order to ensure equal alignment lengths. Sequences harbouring gaps marked as (−) were taken into account using the pairwise deletion option in MEGA X [26]. The final alignments were improved manually and saved in Fasta and Newick formats. Three analytical approaches were used to analyse the sequences.

### 2.3. Distance Analyses and Genetic Diversity

Pairwise distances between nucleotide sequences were calculated as absolute distances [27] using the Kimura 2-parameter model and as LogDet transformations since base composition differed between the sequences [28]. Intraspecific variation and interspecific divergence were assessed in species that are represented by at least two conspecific reference sequences in the dataset [29]. In addition, DNA polymorphism parameters such as the number of haplotypes (h), haplotype diversity (Hd), nucleotide diversity (π), number of segregating sites and the k–mean number of pairwise differences were calculated in DnaSP v5 [30].

### 2.4. Species Delimitation

The final aligned unique haplotype sequences were used for the analysis of species delimitation. Three species delimitation models were applied in order to retrieve the correct species partitioning, namely, the maximum likelihood solution (MPTP), the heuristic solution (SPTP) grouped in the Poisson tree process (PTP) [31] and the assemble species by automatic partitioning (ASAP) [32]. PTP usually uses a phylogenetic tree as input and delimits species using non-ultrametric phylogenies [31]. The maximum likelihood tree was generated using the RAxML software package implemented on a web server ‘‘RAxMLBlackbox’’ (http://embnet.vital-it.ch/raxml-bb/, accessed on 28 November 2021) [33], with a GTR + G substitution model applied in the analyses. The maximum likelihood tree was used as the input file. The analysis was run after the outgroup was removed. ASAP was performed using the command line version downloaded from https://bioinfo.mnhn.fr/abi/public/asap (accessed on 19 November 2021) with default parameters. The calculation methods of Jukes-Cantor (JC69) and Kimura (K80) TS/TV and p-distance methods were tested and compared. Two runs were conducted, one with the whole dataset and the other with independent datasets selected to represent species from the two subfamilies present, i.e., Eristalinae and Syrphinae.

### 2.5. Phylogenetic Analyses

Phylogenetic reconstructions were performed using the neighbour-joining (NJ) [34] and maximum likelihood (ML) approaches [35]. Analyses were run independently with all generated sequences (*n* = 78) and the unique sequences (*n* = 35). In addition to the sequences generated in this study, thirty-nine (39) supplementary sequences of hoverflies were downloaded from Genbank. Identical sequences were removed using DAMBE v.7 [36]. The NJ tree was estimated in MEGAX using the Kimura 2-parameter model of substitution (K2P distance), with 1000 bootstrap replicates. In addition, the dataset was subjected to Bayesian information criterion following the Akaike information criterion corrected (AICc) value and maximum likelihood value (lnL) to select the best model of evolution. Initially, the dataset was partitioned in MEGA X using the find best DNA/protein model option. Confirmation was performed using the web program FindModel (http://hcv.lanl.gov/content/hcv-db/findmodel/findmodel.html, accessed on 19 November 2021), developed from MODELTEST [37]. MEGA X selected the GTR + G + I (first position), GTR + G (second position) and GTR + I (third position) model. FindModel selected the GTR + G (first position), TN93 + G (second position) and HKY + G (third position) models. The ML analysis was performed by taking into consideration the most appropriate models of evolution. Then, the 35 unique sequences along with 39 Genbank sequences were used to construct the ML tree. The South American hoverfly *Alipumilio avispas* Vockeroth, 1964 (AY261709) was used as an outgroup.

## 3. Results

### 3.1. Distance Analyses and Genetic Diversity

DNA sequence analyses indicated that all of the final sequences selected were of good quality. Only 4 out of 78 (5%) sequences studied had missing data. The 78 hoverfly COI sequences represented 15 nominal species from nine genera (Table A1 in Appendix A). More than one conspecific sequence was available for 10 of the species, while the other 5 species were represented by only one barcode. The mean number of barcodes per species was 5.2, with 66.7% of species represented by at least two conspecific sequences (Table A1 in Appendix A). Two distinct COI sequences could not be identified by comparison with the sequences of Jordaens et al. [17]. However, an analysis of previously published COI sequences from Pu et al. [38] confirmed that the two distinct sequences were from *Eupeodes corollae* Fabricius, 1794. The results from the Blast search revealed that a few COI sequences from the species included in this study are available in Genbank from sources other than Jordaens et al. [17].

For both distance calculation methods, the average interspecific distances were 12.50% (3.08–18.09%) and 13.92% (2.86–19.21%), while the average intraspecific distances were 0.35% (0–1.56%) and 0.71 (0–1.97%) for the LogDet and K2P distances, respectively (Table 2 and Table 3). For both distance calculation methods, little variation was observed between the *Phytomia* Guérin-Méneville, 1834 and *Eristalinus* Rondani, 1845 genera, while relatively high variation was observed between the *Phytomia* and the *Syritta* Le Peletier & Serville, 1828 genera (Table 2). The range of distances between *Allograpta nasuta* Macquart, 1842 and *Allograpta fuscotibialis* Macquart, 1842 were low (2.86 and 3.08% for K2P and LogDet distances, respectively) (Table 2). Generally, K2P showed higher pairwise distance values between species than LogDet.

The range of distances within a genus greatly varied with the number of species. The *Allograpta nasuta* sequences showed the highest number of segregating sites and higher pairwise intraspecific LogDet distances compared to other species (Table 3). Very rich haplotype (Hd ˃ 0.95) and nucleotide (π ˃ 0.005) diversities were also recorded in *Eristalinus tabanoides* Jaennicke, 1876. The nucleotide and haplotype diversity values were similar for *Eristalis tenax* Linnaeus, 1758 and *Mesembrius strigilatus* Bezzi, 1912. Additionally, there were three times as many phylogenetically informative sites in *Eristalinus quinquelineatus* Fabricius, 1781 than in the *Allograpta nasuta* Macquart, 1842, *Betasyrphus inflaticornis* Bezzi, 1915 and *Eristalis tenax* Linnaeus, 1758 sequences. Very low distances were observed among the conspecific individuals of *Mesembrius strigilatus* and those of *Phytomia incisa* Wiedemann, 1830 and among conspecific individuals of *Allograpta fuscotibialis* and *Eristalinus tabanoides.* The same trend was observed among the conspecific individuals of *Eristalis tenax, Betasyrphus inflaticornis* and *Eristalinus quinquelineatus,* but not among individuals of *Allograpta nasuta*, where higher mean LogDet distances were observed (Table 3).

No distance divergence was found within the *Eristalinus vicarians* Bezzi, 1915 species. There was no positive relationship between the intraspecific divergence and the number of specimens. When considering the divergences between genera, we observed lower divergence between the genera *Phytomia* and *Eristalinus* (≤10.41%) compared to the relatively high divergence between the other genera (≥14.88%) (Table 4 and Table 5).

The aligned sequences consisted of 35 unique haplotypes (H1–H35). Nucleotide diversity index (π = 0.01423) and the average number of nucleotide differences (k = 8.16) for *A. nasuta* revealed that this species was indeed the most divergent of the local species included in the present study. The majority of species were represented by low frequencies, ranging from 0.00070 to 0.00581, while *E. corollae,* represented by only two specimens, showed relatively high nucleotide diversity (0.00348).

### 3.2. Species Delimitation

We first analysed the ability of the three delimitation models (ASAP, MPTP and SPTP) to determine as accurately as possible the correct number of hoverfly species in our dataset. For each calculation method, ASAP produced 10 partitions with different species numbers; hence, only the partitions with the best ASAP score are presented here (Table 6). The partition with the best ASAP score revealed 14 species in our dataset. However, the partition with the second-best ASAP score exhibited 16 species in our dataset, which was around (±6%) of the expected number of species. The use of the model based on threshold distances led to an estimate of 14 hoverflies species (confidence interval: 14–17) for our whole dataset. MPTP and SPTP were not congruent in validating the 14 species as in ASAP. In contrast, for the Syrphinae dataset, both models revealed 5 species (MPTP model) and 6 species (SPTP model), while they both revealed 10 species for the Eristalinae dataset, bringing the total number of species to 15 (MPTP model) and 16 (SPTP model). The SPTP model agreed with the results of the ASAP analysis partitioning with the second-best ASAP score, which also identified 10 and 6 species for the Eristalinae and Syrphinae subfamilies, respectively.

### 3.3. Phylogenetic Analyses

**Figure 2:**
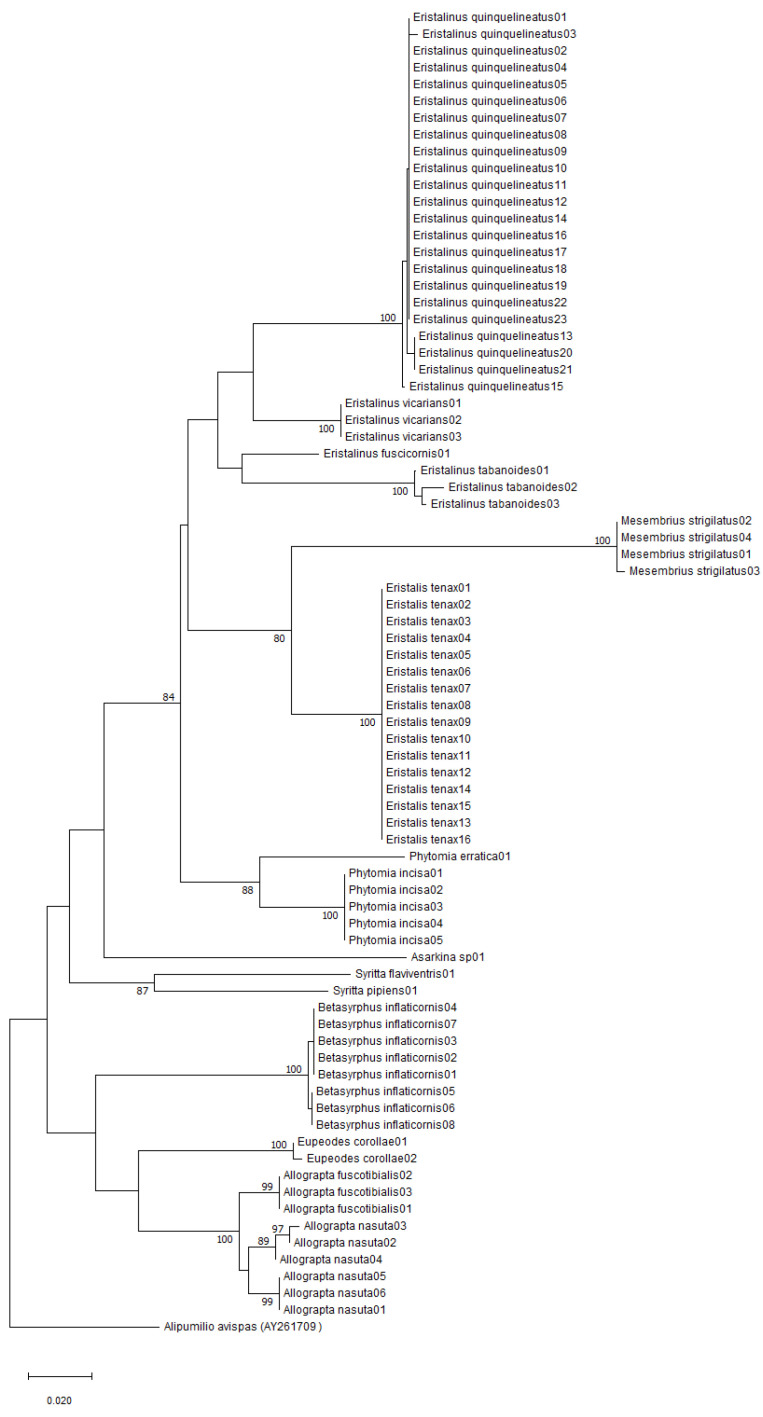
Neighbour-joining tree depicting the degree of relationship between the Afrotropical hoverfly species produced from the COI gene. Bootstrap support values higher than 70 are reported on the tree. Information on specimens is given in the Table A1 in Appendix A. *Alipumilio avispas* (AY261709) was included as the outgroup.

**Figure 3:**
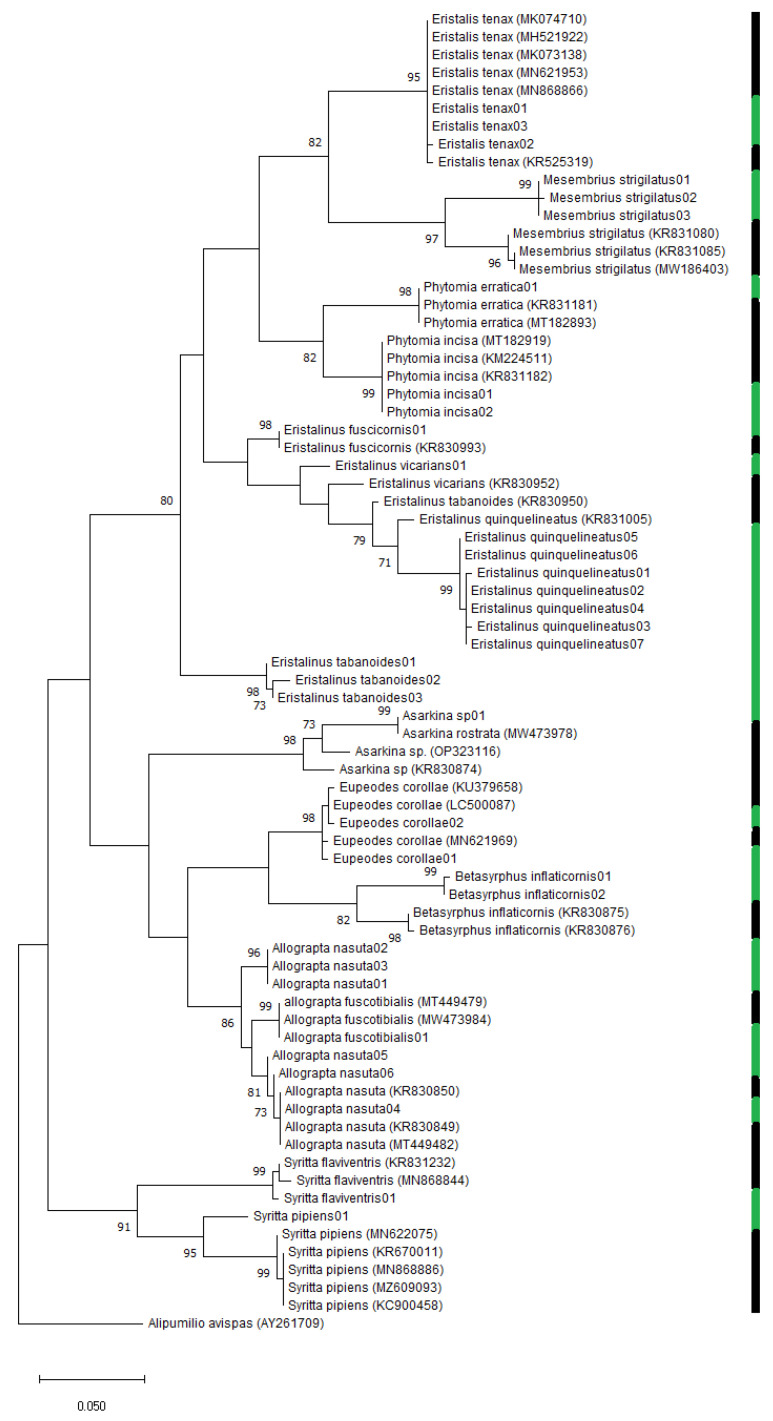
Maximum likelihood tree based on the general time-reversible model. Bootstrap support values higher than 70 are reported on the tree. The twelve green bars represent specimens included in species delimitation models (ASAP, MPTP and SPTP). The thirteen black bars indicate published sequences (and accession numbers) downloaded from either Genbank or Bold. *Alipumilio avispas* (AY261709) was included as the outgroup.

The neighbour-joining (NJ) analysis of the COI sequences revealed that all species formed distinct clusters in the tree (Figure 2). All nine species represented by more than two haplotypes formed clusters with 100% bootstrap values, except *A. nasuta* where the combined bootstrap value of the clusters was 95% (Figure 2). However, a Genbank sequence putatively identified as *Allograpta fuscotibialis* (Accession number: MT449479) grouped closely with sequences of *Allograpta nasuta* identified with the help of the published work of Jordaens et al. [17]. The distances between the two taxa ranged from 2.86 to 3.08%, and the grouping of that putative *A. fuscotibialis* sequence with the rest of the known *A. nasuta* sequences was supported by a bootstrap value of 96 (Figure 3).

In the maximum likelihood (ML) analysis with species with more than two haplotypes, four nominal species (44.44%) were supported by high bootstrap values (˃98%). A low (78%) bootstrap value supported the grouping of *E. quinquelineatus* sequences with the putative barcode of *E. quinquelineatus* from Genbank (Accession number: KR831005) (Figure 3). The barcodes of the genus *Allograpta* formed two clusters, and one of these included a Genbank barcode of *Allograpta nasuta* (Accession number: KR830850). The ML tree suggested a close relationship between *Allograpta nasuta* and *Allograpta fuscotibialis* followed by *Mesembrius strigilatus* and *Eristalis tenax*, *Syritta pipiens* Linnaeus, 1758 and *Syritta flaviventris* Macquart, 1842 and *Phytomia erratica* Bezzi, 1912 and *Phytomia incisa* Wiedemann, 1830. The divergence within the genus *Eristalinus* was observed in the tree, as three distinct clusters were formed for the species in this genus (Figure 3).

## 4. Discussion

The COI gene has been used successfully for species delimitation in Afrotropical hoverfly genera such as *Merodon* Meigen, 1803 [21,22], *Sphaerophoria* Le Peletier & Serville, 1828 [20], *Eumerus* Meigen, 1803 [18,19], *Toxomerus* Fabricius, 1798 [39], *Allograpta* Osten Sacken, 1875, *Asarkina* Macquart 1842, *Episyrphus* Matsumura, 1917 and *Exallandra* Vockeroth, 1969 [24]. Using the COI barcode, Jordaens et al. [17] identified 90 species from 24 genera collected in Ghana, Togo, Benin and Nigeria. Even though species estimation from the Afrotropics is approximately 600 species, their study helped to improve species identification in this understudied group of hoverflies. Our study is the first local attempt using DNA barcoding to ascertain the taxonomy and establish the genetic richness and differentiation of species from the Free State province of South Africa. The three analytical approaches used in the present study conformed to morphotype assignment, except for the *Allograpta* genus where doubt still exists with regard to the number of species represented within the genus. In modern taxonomy, the use of DNA barcoding in combination with traditional taxonomy has led to the creation of reference libraries (of major groups of living organisms), which are useful for congruent species delimitation as in the case of Afrotropical hoverflies (e.g., Mengual et al. [20]).

In the present study, the different species delimitation methods tested resulted in various numbers of species, thus demonstrating the challenge of applying adequate criteria for distinguishing between species. The method named assemble species by automatic partitioning (ASAP), developed by Puillandre et al. [32], estimated a range of 14 to 17, while the Poisson tree processes based on the MPTP and SPTP [31] models estimated 15 and 16 species. In spite of the difference in species numbers, the results from the three models were closer when we divided the whole dataset into two subsets according to the subfamilies (Eristalinae and Syrphinae). The two subsets differed in terms of species, sequence and haplotype numbers. The number of sequences for each dataset was 66 and 12 for the Eristalinae and the Syrphinae datasets, respectively. The MPTP and SPTP models recovered the same species number for the Eristalinae dataset, confirming the usefulness of models based on the phylogenetic species concept (PSC) for species delimitation in this group. The PSC proposed by Cracraft [40] defined a species as “the smallest diagnosable cluster of individual organisms within which there is a parental pattern of ancestry and descent”. Although it has been recently criticised because it oversplits the number of taxa [41,42,43], the use of such a concept in conjunction with another model based on genetic distance calculations appears to be useful for determining the true number of species.

In the present study, the COI divergence between *A. nasuta* and *A. fuscotibialis* was low (mean K2P = 2.86%) and comparable to that found between other Afrotropical hoverfly species in the genus *Melanostoma* (*M.* cf. *floripeta* Speiser, 1910 and *M.* cf. *bituberculatum* Loew, 1858, mean K2P = 2.95%) and the genus *Microdon* (*Microdon brevicornis* Loew, 1858 and *Archimicrodon* sp., Hull, 1937 mean K2P = 2.84%) [14]. From the study of Jordaens et al. [17], divergences between species from monophyletic genera were even lower for the genus *Allobaccha* (*A. euryptera* Bezzi, 1908 and *A. picta* Wiedemann, 1830, mean K2P = 0.51%) and the genus *Rhingia* (*R. caerulescens* Loew, 1858 and *R. semicaerulea* Austen, 1893, mean K2P = 1.66%), whereas high intraspecific divergences were observed in *Graptomyza triangulifera* Bigot, 1883 (mean K2P = 2.13%), *Asarkina ericetorum* Fabricius, 1781 (mean K2P = 2.63%), *Phytomia natalensis* Macquart, 1850 (mean K2P = 2.93%), *Allobaccha picta* Wiedemann, 1830 (mean K2P = 3.07%), *Syritta bulbus* Walker, 1849 (mean K2P = 4.57%) and *Polybiomyia divisa* Walker, 1857 (mean K2P = 5.63%). One of the explanations provided by the authors is that this may reflect geographical structuring or evolutionary history and that speciation events were recent in these lineages; consequently, the COI sequences had not yet accumulated many mutations. Thus, they argued that this could signal the occurrence of cryptic species. In our case, the values for intraspecific variations were consistently lower than those for interspecific divergence. This is in accordance with the findings from other studies carried out in restricted geographical zones on other taxa including earthworms [44] and bivalves [45]. Regarding the two taxa of *Allograpta* (Table 2)*,* without deep morphological investigation, it is difficult to use the present distances to sort out the ambiguity between them. The use of additional molecular markers (e.g., nuclear 28S and 18S ribosomal RNA genes) could also provide more insights into speciation dynamics in these organisms [20]. The use of additional markers can increase the power of taxonomic resolution or provide the opportunity to test different hypotheses because different markers would potentially have different evolutionary histories as they evolve differently (slow and fast rates of mutation). For instance, the 14 species recovered with the ASAP model could correspond to a relatively low rate of speciation, but when the speciation rate increases it becomes difficult for the model to delimit species by using pairwise genetic differences. Indeed, a shortcoming of this model is that individuals that experienced recent speciation events with faster speciation rates (˃1) could be wrongfully considered as the same species [32]. Also, the fact that certain taxa, such as *E. fuscicornis*, *P. erratica*, *S. flaviventris*, *S. pipiens* and *Asarkina* sp. Macquart, 1842 (Table A1 in Appendix A) were represented by a limited number of sequences might have also influenced species delimitation. Ahrens et al. [46] discussed the issue of singletons (lone representative sequence of a species) in DNA-based species delimitation studies and argued that a high proportion of singletons may compromise the estimation of intra- versus interspecies evolutionary processes. From their study on approximately 100 species of beetles (Scarabaeidae family), 48.5% of species were singletons, which produced poor results when different delimitation approaches were used among which were the Poisson tree processes (PTP), generalized mixed yule coalescent (GMYC) and automatic barcode gap discovery (ABGD). Given our relatively small dataset, the same limitation might have applied as a result of the overall percentage of singletons, which stood at 33.33%.

Based on Blast analysis, all of our sequences showed similarity to published sequences in Genbank within a range of 92.48–100%. All relevant Genbank sequences included in the dataset were genetically distant, with 0 to 7.85% divergence among the 15 putative Afrotropical species. Taxa clustered together with high bootstrap values irrespective of their number of sequences in the dataset. The neighbour-joining tree exhibited 100% bootstrap values for all clusters, except in *A. nasuta* where a 95% value was observed. In the case of *A. nasuta* and *A. fuscotibialis,* the occurrence of high intraspecific molecular variability for the COI marker (as shown in Table 3) indicates that this marker may not be adequate for distinguishing between species within the *Allograpta* genus. This was further supported by the fact that both taxa were conflated in the tree with significant bootstrap support values. Throughout the literature, different cases of inconsistency between morphological and molecular data have occurred (e.g., for *Allograpta* lineages, Mengual et al. [24]).

The data generated from this study support the identification of Afrotropical hoverflies for 15 nominal species from nine genera. Moreover, based on the work of Whittington [2], who assessed the faunal diversity of Afrotropical Syrphidae, 196 Syrphidae species occurring in South Africa were compiled into a checklist (see details here: https://www.syrphidae.com/checklist.php?country=ZA, accessed on 17 January 2023. Our work reveals that at least 15 different species belonging to this family of Diptera are found in our study area. Interestingly, five of these species, namely, *Betasyrphus inflaticornis*, *Mesembrius strigilatus*, *Eristalinus tabanoides*, *Eristalinus vicarians* and *Eristalinus fuscicornis* (see Table A1 in Appendix A), were not listed in the checklist of Whittington [2]. This could imply that this author had overlooked (or misidentified certain species) or that over the last 20 years, the distribution dynamics of Afrotropical Syrphidae have significantly changed and previous species not reported in South Africa were introduced into the country. When compared with the finding of Jordaens et al. [17], who used DNA barcoding to identify 90 hoverfly species distributed across four countries in west Africa, we found the following overlapping species (i.e., which also occur in South Africa): *Phytomia incisa*, *Eristalinus vicarians*, *Eristalinus tabanoides*, *Betasyrphus inflaticornis*, *Mesembrius strigilatus*, *Allograpta nasuta*, *Syritta flaviventris*, *Asarkina* sp. and *Phytomia erratica.*

Despite its utility, however, DNA barcoding is not a panacea for the study of biodiversity. An integrative taxonomical approach including physiological and ecological features must be prioritised (e.g., Aguado-Aranda et al. [13]; Porco et al. [47]) since it provides additional data useful for separating cryptic diversity even in common species.

With the barcodes generated in this study, the identification of Afrotropical species could be improved, knowing that about 40% of the known species cannot be identified using the available identification keys [2]. Currently, more than 90% of the available Afrotropical COI sequences are from the same source, viz. Jordaens et al. [17]. However, these authors mainly focused on species from West Africa. The present study contributes by adding to the available hoverfly DNA barcode data and by confirming the geographic distribution of some of the Afrotropic species belonging to this important group of flies.

## Figures and Tables

**Table 1:** Primers used for amplifying the COI gene in this study.

Gene Region	Primer	Sequence	Source
COI	LCO1490	5′-GGTCAACAAATCATAAAGATATTGG-3′	Folmer et al. [25]
	HCO2198	5′-TAAACTTCAGGGTGACCAAAAAATCA-3′	Folmer et al. [25]

**Table 2:** Mean pairwise interspecific distance (%) between the Afrotropical hoverfly species sampled in the study area. Kimura 2-parameter distances are shown in the lower left matrix, and LogDet distances are shown in the upper right matrix.

	1	2	3	4	5	6	7	8	9	10	11	12	13	14	15
1 *Betasyrphus inflaticornis*		15.25	9.03	15.66	14.14	13.19	14.83	13.45	12.54	11.01	11.01	13.42	15.38	14.35	13.80
2 *Eristalis tenax*	16.93		15.88	10.19	14.53	10.18	9.51	14.26	15.10	15.57	15.57	9.71	9.83	12.10	9.47
3 *Eupeodes corollae*	10.05	17.81		17.25	13.70	12.76	13.44	13.62	13.94	8.02	8.34	11.39	15.17	11.58	12.44
4 *Mesembrius strigilatus*	17.17	11.45	19.16		17.37	14.82	13.39	18.09	16.52	15.83	15.64	13.28	14.27	12.80	13.53
5 *Asarkina* sp.	15.84	15.84	15.20	18.93		14.40	15.78	15.23	12.68	12.37	12.88	12.71	12.84	14.86	11.66
6 *Phytomia erratica*	14.57	11.26	14.56	16.30	15.84		6.78	11.87	11.57	14.99	14.90	8.92	11.07	9.84	10.68
7 *Phytomia incisa*	6.07	10.07	14.99	14.59	16.93	6.94		14.00	14.50	15.34	14.44	9.24	9.86	9.75	11.53
8 *Syritta flaviventris*	14.78	15.43	15.63	19.21	16.93	13.11	15.03		9.52	14.52	15.60	13.21	14.82	14.31	13.62
9 *Syritta pipiens*	14.35	16.71	16.50	18.27	14.35	13.10	16.07	11.05		11.88	12.70	11.82	13.24	13.11	12.86
10 *Allograpta fuscotibialis*	12.68	17.59	9.45	17.81	13.93	16.71	16.72	16.28	14.15		3.08	10.86	12.10	12.26	11.21
11 *Allograpta nasuta*	12.68	17.37	9.65	17.58	14.14	16.71	16.06	17.17	14.78	2.86		10.92	12.10	11.29	11.79
12 *Eristalinus fuscicornis*	14.99	10.65	13.10	14.79	14.14	10.25	10.45	14.37	13.72	12.27	12.48		6.72	7.04	4.95
13 *Eristalinus quinquelineatus*	16.93	11.05	16.93	15.45	14.14	12.48	11.06	16.28	14.78	13.31	13.94	7.13		9.95	6.08
14 *Eristalinus tabanoides*	16.06	13.11	13.52	13.95	16.49	11.05	10.86	15.24	14.57	14.14	13.10	8.08	10.68		9.40
15 *Eristalinus vicarians*	15.41	10.65	14.35	14.80	12.89	12.27	12.70	15.20	14.78	12.68	13.31	5.24	6.38	10.27	

**Table 3:** Mean intraspecific variation (%) for each species sampled in the study area. Only species represented by more than one specimen were used for the analysis. *n* = number of specimens. Standard errors are presented in parentheses.

Species	*n*	Number of Segregating Sites (S)	Number of Haplotype (h)	Haplotype Diversity (Hd)	Nucleotide Diversity (π)	k–Average Number of Differences (SE)	Number of Parsimony Informative Sites	Mean LogDet Distance (%) (SE)
*Phytomia incisa*	5	1	2	0.4000	0.00070	0.40 (0.22)	0	0.07 (0.07)
*Eristalinus quinquelineatus*	23	6	7	0.5217	0.00145	0.83 (0.23)	3	0.15 (0.07)
*Eristalis tenax*	16	1	2	0.5000	0.00087	0.50 (0.33)	1	0.09 (0.08)
*Betasyrphus inflaticornis*	8	1	2	0.5357	0.00093	0.53 (0.37)	1	0.10 (0.10)
*Mesembrius strigilatus*	4	1	2	0.5000	0.00087	0.50 (0.33)	0	0.06 (0.06)
*Allograpta nasuta*	6	16	4	0.8000	0.01423	8.16 (2.06)	1	1.56 (0.39)
*Eupeodes corollae*	2	2	2	1.0000	0.00348	2 (1.11)	0	0.40 (0.28)
*Eristalinus tabanoides*	3	5	3	1.0000	0.00581	3.33 (1.49)	0	0.58 (0.26)
*Eristalinus vicarians*	3	0	1	0.0000	0	0 (0)	0	0 (0)
*Allograpta fuscotibialis*	3	4	3	1.0000	0.00467	2.66 (1.31)	0	0.52 (0.26)

**Table 4:** Mean variation (%) within each genus. Only genera represented by more than one species were used for the analysis. *n* = number of species.

Genus	*n*	Average Number of Differences (k)	SE	Mean LogDet Distance (%)	SE
*Phytomia*	2	38	1.25	7	1.14
*Eristalinus*	4	34.33	1.30	5.92	0.89
*Syritta*	2	59	1.25	9.50	1.26
*Allograpta*	2	16	2.61	2.85	0.63

**Table 5:** Mean divergence between genera given as LogDet distances (%).

	*Phytomia*	*Eristalinus*	*Syritta*	*Allograpta*
*Phytomia*				
*Eristalinus*	10.41 (1.40)			
*Syritta*	12.99 (1.60)	13.26 (1.60)		
*Allograpta*	14.88 (1.71)	11.50 (1.40)	13.05 (1.62)	

**Table 6:** Results of the analysis of the sequence dataset based on delimitation models.

Subfamily	Number of Sequences	Expected Number of Species	Number of Haplotypes	Predictions of Delimitation Models		
				ASAP	MPTP	SPTP
Eristalinae	66	10	21	10	10	10
Syrphinae	12	5	12	6	5	6

## Data Availability

The data presented in this study are openly available in Genbank at https://www.ncbi.nlm.nih.gov/genbank/ (accessed on 15 July 2023), reference number [OL981963—OL982040].

## Appendix A

**Table A1:** List of Afrotropical hoverflies (n = 78) used for COI sequencing in this study.

Top Species Match	Sampling Location	Latitude/Longitude	Sampling Date	Collector	Code in Alignment	Genbank Accession Number
*Allograpta nasuta* (Macquart, 1842)	Free State: Harrismith	−28.2756/29.1226 −28.276/29.1227 −28.276/29.1227 −28.276/29.1227 −28.276/29.1227 −28.276/29.1227	4 May 2018 4 May 2018 4 May 2018 22 April 2018 2 May 2018 22 April 2018	M. Kamdem	*A. nasuta 01* *A. nasuta 02* *A. nasuta 03* *A. nasuta 04* *A. nasuta 05* *A. nasuta 06*	OL981965 OL981966 OL981967 OL981968 OL981969 OL981970
*Allograpta fuscotibialis* (Macquart, 1842)	Free State: Harrismith Free State: Golden Gate	−28.2756/29.1238 −28.2756/29.1226 −28.5084/28.6159	19 March 2018 11 March 2018 26 April 2018	M. Kamdem	*A. fuscotibialis 01* *A. fuscotibialis 02* *A. fuscotibialis 03*	OL982040 OL981963 OL981964
*Asarkina* sp. (Macquart, 1842)	Free State: Phuthaditjhaba	−28.4861/28.8248	9 March 2018	M. Kamdem	*Asarkina* sp. *01*	OL981973
*Betasyrphus inflaticornis* (Bezzi, 1915)	Free State: Verkykerskop Free State: Phuthaditjhaba Free State: Harrismith	−27.9921/29.18 −28.4861/28.8248 −28.4861/28.8248 −28.2756/29.1238 −28.2756/29.1238 −28.276/29.1227 −28.2843/29.1159 −28.2843/29.1159	4 May 2018 9 March 2018 19 March 2018 21 March 2018 21 March 2018 21 March 2018 12 April 2018 12 April 2018	M. Kamdem	*B. inflaticornis 01* *B. inflaticornis 02* *B. inflaticornis 03* *B. inflaticornis 04* *B. inflaticornis 05* *B. inflaticornis 06* *B. inflaticornis 07* *B. inflaticornis 08*	OL982032 OL982033 OL982034 OL982035 OL982036 OL982037 OL982038 OL982039
*Eristalinus fuscicornis* (Karsch, 1887)	Free State: Harrismith	−28.2756/29.1226	13 March 2018	M. Kamdem	*E. fuscicornis 01*	OL982026
*Eristalinus tabanoides* (Jaennicke, 1876)	Free State: Harrismith Free State: Phuthaditjhaba	−28.2741/29.1253 −28.4861/28.8248 −28.4861/28.8248	12 April 2018 22 March 2018 17 April 2018	M. Kamdem	*E. tabanoides 01* *E. tabanoides 02* *E. tabanoides 03*	OL982027 OL982028 OL982029
*Eristalinus quinquelineatus* (Fabricius, 1781)	Free State: Phuthaditjhaba Free State: Verkykerskop Free State: Harrismith	−28.4861/28.8248 −28.4861/28.8248 −28.4861/28.8248 −28.45/28.88 −28.45/28.88 −28.4861/28.8248 −27.9921/29.18 −28.2687/29.1325 −28.276/29.1227 −28.2668/29.1344 −28.276/29.1227 −28.2793/29.1191 −28.2684/29.1333 −28.2756/29.1238 −28.2741/29.1253 −28.2687/29.1325 −28.2687/29.1325 −28.2756/29.1226 −28.2687/29.1325 −28.2793/29.1191 −28.2793/29.1191 −28.276/29.1227 −28.2684/29.1333	25 March 2018 25 March 2018 7 April 2018 4 May 2018 9 April 2018 2 May 2018 16 May 2018 22 April 2018 02 May 2018 2 May 2018 17 April 2018 21 May 2018 21 May 2018 12 April 2018 12 April 2018 12 April 2018 6 March 2018 18 May 2018 21 May 2018 06 March 2018 06 March 2018 24 March 2018 7 May 2018	M. Kamdem	*E. quinquelineatus 01* *E. quinquelineatus 02* *E. quinquelineatus 03* *E. quinquelineatus 04* *E. quinquelineatus 05* *E. quinquelineatus 06* *E. quinquelineatus 07* *E. quinquelineatus 08* *E. quinquelineatus 09* *E. quinquelineatus 10* *E. quinquelineatus 11* *E. quinquelineatus 12* *E. quinquelineatus 13* *E. quinquelineatus 14* *E. quinquelineatus 15* *E. quinquelineatus 16* *E. quinquelineatus 17* *E. quinquelineatus 18* *E. quinquelineatus 19* *E. quinquelineatus 20* *E. quinquelineatus 21* *E. quinquelineatus 22* *E. quinquelineatus 23*	OL982000 OL982001 OL982002 OL982003 OL982004 OL982005 OL982006 OL982007 OL982008 OL982009 OL982010 OL982011 OL982012 OL982013 OL982014 OL982015 OL982016 OL982017 OL982018 OL982019 OL982020 OL982021 OL982022
*Eristalinus vicarians* (Bezzi, 1915)	Free State: Phuthaditjhaba	−28.4861/28.8248 −28.4861/28.8248 −28.4861/28.8248	30 March 2018 16 April 2018 04 May 2018	M. Kamdem	*E. vicarians 01* *E. vicarians 02* *E. vicarians 03*	OL982023 OL982024 OL982025
*Eristalis tenax* (Linnaeus, 1758)	Free State: Harrismith Free State: Phuthaditjhaba	−28.2672/29.1375 −28.276/29.1227 −28.2668/29.1344 −28.2741/29.1253 −28.2684/29.1333 −28.2849/29.1169 −28.2672/29.1375 −28.45/28.88 −28.45/28.85 −28.4861/28.8248 −28.4861/28.8248 −28.4861/28.8248 −28.4861/28.8248 −28.4861/28.8248 −28.45/28.88 −28.4861/28.8248	2 March 2018 6 March 2018 24 March 2018 21 April 2018 21 May 2018 7 May 2018 12 May 2018 14 April 2018 14 April 2018 3 May 2018 21 April 2018 16 March 2018 4 May 2018 4 May 2018 4 May 2018 23 May 2018	M. Kamdem	*E. tenax 01* *E. tenax 02* *E. tenax 03* *E. tenax 04* *E. tenax 05* *E. tenax 06* *E. tenax 07* *E. tenax 08* *E. tenax 09* *E. tenax 10* *E. tenax 11* *E. tenax 12* *E. tenax 13* *E. tenax 14* *E. tenax 15* *E. tenax 16*	OL981984 OL981985 OL981986 OL981987 OL981988 OL981989 OL981990 OL981991 OL981992 OL981993 OL981994 OL981995 OL981996 OL981997 OL981998 OL981999
*Eupeodes corollae* (Fabricius, 1794)	Free State: Verkykerskop	−27.9921/29.18 −27.9921/29.18	4 May 2018 4 May 2018	M. Kamdem	*E. corollae 01* *E. corollae 02*	OL982030 OL982031
*Mesembrius strigilatus* (Bezzi, 1912)	Free State: Harrismith	−28.276/29.1227 −28.2756/29.1238 −28.2756/29.1226 −28.2756/29.1226	17 March 2018 29 March 2018 22 April 2018 22 April 2018		*M. strigilatus 01* *M. strigilatus 02* *M. strigilatus 03* *M. strigilatus 04*	OL981980 OL981981 OL981982 OL981983
*Phytomia erratica * (Bezzi, 1912)	Free State: Phuthaditjhaba	−28.4861/28.8248	17 March 2018	M. Kamdem	*P. erratica 01*	OL981974
*Phytomia incisa * (Wiedemann, 1830)	Free State: Golden Gate Free State: Phuthaditjhaba	−28.5084/28.6159 −28.4861/28.8248 −28.45/28.88 −28.4861/28.8248 −28.4861/28.8248	24 April 2018 21 April 2018 18 April 2018 13 March 2018 13 March 2018	M. Kamdem	*P. incisa 01* *P. incisa 02* *P. incisa 03* *P. incisa 04* *P. incisa 05*	OL981975 OL981976 OL981977 OL981978 OL981979
*Syritta flaviventris* (Macquart, 1842)	Free State: Harrismith	−28.276/29.1227	19 March 2018	M. Kamdem	*S. flaviventris 01*	OL981971
*Syritta pipiens * (Linnaeus, 1758)	Free State: Harrismith	−28.276/29.1227	22 April 2018	M. Kamdem	*S. pipiens 01*	OL981972
